# Acute Autonomic, Sensory and Motor Neuropathy Associated with Meningoencephalitis

**DOI:** 10.4137/ccrep.s2194

**Published:** 2009-02-12

**Authors:** Satoko Kinoshita, Kazuma Sugie, Hiroshi Kataoka, Miho Sugie, Makito Hirano, Satoshi Ueno

**Affiliations:** 1 Department of Neurology, Nara Medical University School of Medicine, Nara, Japan.; 2 Department of Neurology and Rehabilitation Medicine, Nara Prefectural Rehabilitation Center, Nara, Japan.

**Keywords:** acute autonomic, motor and sensory neuropathy (AASMN), intravenous immunoglobulin (IVIg), meningoencephalitis, dysautonomia

## Abstract

We report the first case of acute autonomic, motor and sensory neuropathy (AASMN) associated with meningoencephalitis. A 62-year-old man presented with fever, neck stiffness, and coma. Respiratory failure developed. Magnetic resonance images showed an abnormality in the medial temporal lobe. Cerebrospinal fluid analysis revealed pleocytosis with a high protein level. Intensive care gradually improved the consciousness level, but paralysis of the four extremities persisted. Nerve conduction studies revealed demyelinating sensory and motor polyneuropathy. Severe orthostatic hypotension, urinary retention, and constipation were also present. Clinical autonomic tests suggested both sympathetic and parasympathetic dysfunction. After intravenous immunoglobulin therapy, motor and sensory symptoms resolved rapidly; dysautonomia resolved gradually over the next 2 months. The response to immunological therapy and the presence of antecedent infection suggest that AASMN is a postinfectious, immune-mediated, autonomic, sensory and motor nervous system dysfunction.

## Introduction

Acute autonomic, motor and sensory neuropathy (AASMN) is a relatively new clinical entity and a rare neuromuscular disorder, characterized by prominent dysautonomia with somatic motor and sensory involvement.^1^ AASMN has been categorized as a variant form of acute autonomic and sensory neuropathy or as Guillain-Barré syndrome (GBS) with dysautonomia.^1^ Although the pathogenesis of AASMN remains unclear, most patients have antecedent infection.^1^–^3^ Such infection usually involves the upper respiratory and gastrointestinal systems; meningoencephalitis has not been reported.

We describe a man in whom AASMN developed after acute encephalitis. He received intravenous immunoglobulin (IVIg) therapy, and the motor and sensory disturbances resolved rapidly, followed by a gradual improvement in dysautonomia. We believe that this is the first documented case of AASMN associated with meningoencephalitis.

## Case Report

**Figure 1:**
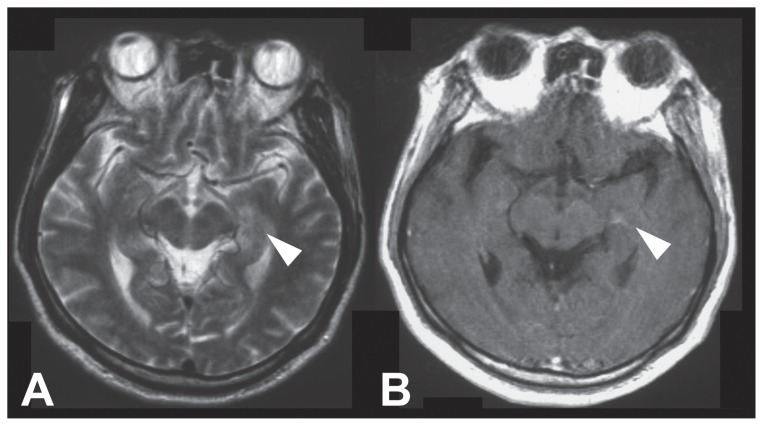
Brain magnetic resonance imaging. **A**) A T2-weighted image, showing increased signal intensity in the left medial temporal lobe. **B**) A contrast-enhanced T1 sequence, showing abnormal enhancement in the same area. After intensive care, these abnormal signals disappeared.

A 62-year-old man had fever, headache, general malaise, and bilateral swelling of the parotid glands. Three days after symptom onset, he was admitted to our hospital because of impaired consciousness. On admission, the patient was drowsy. Neurologic examinations revealed nuchal stiffness, but muscle strength and deep tendon reflexes were normal. Plantar responses were flexor. The pupils were equal and normally reactive to light. No cerebellar symptoms were present. Laboratory examinations revealed mild thrombocytopenia and hyponatremia. Serum antibodies against herpes simplex, varicella-zoster, Epstein-Barr, and mumps viruses, cytomegalovirus, and human immunodeficiency virus (HIV) were not elevated. Cerebrospinal fluid (CSF) analysis revealed pleocytosis (70/μl, predominantly lymphocytes) with a markedly increased protein level (526 mg/dl). Herpes simplex virus DNA was not detected in CSF. Magnetic resonance images (MRI) of the brain revealed an abnormal signal in the left medial temporal lobe (Fig. 1). There were no epileptiform discharges on the electroencephalogram. Several days later, the patient became comatose and received artificial ventilation because of respiratory failure with hypercapnia. His consciousness gradually improved after intravenous administration of aciclovir, cytarabine, and methylprednisolone (1 g/day for 3 days). One month after symptom onset, he became alert, and repeated CSF examinations revealed no leukocytes, with a normal protein level (56 mg/dl). However, paralysis of the arms and legs developed, and the deep tendon reflexes were diminished. Sensory examinations demonstrated diminished pain sensation in the distal part of the legs and diminished vibration sense in the arms and legs. It was difficult to precisely determine the time of the onset of muscle weakness and sensory disturbance because the patient was sedated with midazolam and receiving ventilatory support. Nerve conduction studies revealed demyelinating sensory and motor polyneuropathy (Table 1). The serum contained no anti-ganglioside antibodies. The blood pressure was 143/97 mmHg in the supine position and 73/49 mmHg on 70-degree passive head-up tilt; the heart rate was 82 bpm and 86 bpm, respectively, suggesting orthostatic hypotension (OH). He also had urinary retention and constipation. The R-R interval variation at rest was decreased. On 123I-metaiodobenzylguanidine (MIBG) myocardial scintigraphy, the MIBG washout rate was slightly decreased (42.2%), indicating mild cardiac sympathetic involvement. Intraocular instillation of a 1.25% epinephrine solution provoked hypersensitive mydriasis, suggesting postganglionic autonomic failure.

**Figure 2:**
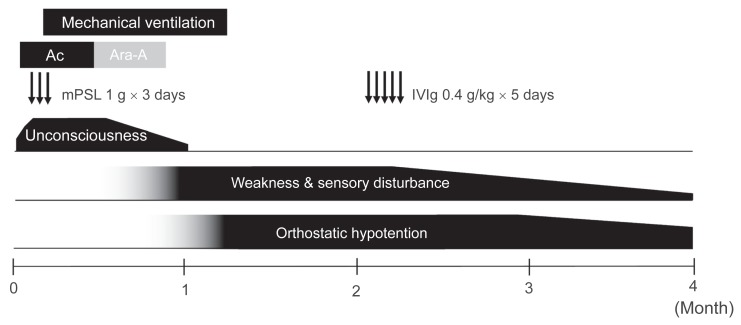
Clinical course. **Abbreviations:** mPSL, methylprednisolone; Ac, acyclovir; Ara-A, vidarabine; IVIg, intravenous immunoglobulin.

The results of these clinical, laboratory, electro-physiologic, and imaging studies indicated somatic motor and sensory demyelinating neuropathy accompanied by sympathetic and parasympathetic dysfunction, findings consistent with AASMN. The patient was given IVIg at a dose of 0.4 g/kg body weight/day for 5 days. Motor and sensory symptoms resolved markedly and promptly within 2 months (Fig. 2). Deep tendon reflexes in the arms and legs continued to be weak. Repeated nerve conduction studies revealed slight improvements in motor and sensory nerve conduction velocities in the upper and lower extremities. OH, urinary disturbance, and constipation resolved gradually over the course of the next 2 months.

## Discussion

We report here the first case of AASMN associated with antecedent meningoencephalitis. Only nine patients with AASMN have been reported previously.^1^–^8^ Antecedent infection has included upper respiratory tract infection, gastrointestinal illness, or high-grade fever. Postmeningoencephalitic dysautonomia has sporadically been reported.^9^–^11^ Increasing evidence suggests that postmeningoencephalitic dysautonomia is a variant form of GBS caused by an immunologic defect. The dysautonomia in our patient responded to treatment with IVIg, as seen as in GBS.^12^ Similar therapeutic effects of IVIg in dysautonomia and GBS suggest the involvement of common elements in the pathogenesis of these two postinfectious diseases.

Autonomic nervous system dysfunction can be either central or, more commonly, peripheral in origin.^9^,^10^ In our patient, the results of MIBG myocardial scintigraphy and intraocular instillation of an epinephrine solution indicated postganglionic autonomic failure. Brain MRI revealed no abnormality in the hypothalamus or brainstem. Collectively, the clinical course and the results of neurophysiologic and autonomic nervous system studies suggested peripheral autonomic involvement rather than central origin.

Our patient responded to IVIg therapy, with rapid, almost complete resolution of motor and sensory disturbances, followed by a gradual improvement in dysautonomia, similar to one previous report.^3^ However, most previous patients with AASMN received corticosteroid therapy, plasmapheresis, or both. These treatments were not very effective for autonomic and sensory disturbances: only 33% of patients had marked resolution, whereas motor symptoms resolved completely in 67% of patients. These results suggest that the autonomic and sensory disturbances were essentially intractable. Although our experience is limited to only two cases (one previous case in addition to the present one), our findings suggest that IVIg is an important treatment option for this disease. The delayed response of dysautonomia to IVIg therapy may have been ascribed to the fact that unmyelinated autonomic nerve fibers are more vulnerable than myelinated somatic nerve fibers.

Although the pathogenesis of AASMN remains unclear, the presence of antecedent infection, increased protein levels in CSF, and a response to immunologic therapy suggest that AASMN is a postinfectious, immune-mediated autonomic, sensory and motor nervous system dysfunction.

## Figures and Tables

**Table 1:** Summary of the results of nerve conduction studies.

	Median N	Ulnar N	Tibial N	Peroneal N	Sural N
DL (ms)	3.8 (2.9–4.0)	3.3 (2.2–3.4)	5.7 (3.6–6.6)	NR (3.9–6.8)	
MCV (m/s)	47.0 (51.8–65.6)	44.0 (52.2–67.8)	39.4 (40.4–53.9)	NR (43.0–57.5)	
CMAP (mV)	9.9 (10.0–23.0)	7.4 (10.0–21.0)	3.0 (9.0–35.0)	NR (1.0–10.0)	
FWL (ms)	32.1 (22.0–32.0)	33.0 (24.0–32.0)	NR (41.0–51.0)	NR (43.0–53.0)	
SCV (m/s)	46.1 (50.0–66.6)	NR (51.4–62.3)			39.4 (42.1–67.8)
SNAP (mV)	6.0 (1.0–25.0)	NR (0.2–18.0)			7.3 (1.0–23.0)

**Abbreviation:** NR, no response obtained; DL, distal latency; MCV, motor conduction velocity; CMAP, compound muscle action potential; FWL, F wave latency; SCV, sensory conduction velocity; SNAP, sensory nerve action potential.

**Note:** Normal ranges are in parentheses.
